# Strain and Temperature Sensing Based on Different Temperature Coefficients fs-FBG Arrays for Intelligent Buoyancy Materials

**DOI:** 10.3390/s24092824

**Published:** 2024-04-29

**Authors:** Meng Tian, Minggan Lou, Wei Zhang, Wenzhu Huang, Kaiqi Yan, Bin Liao, Wentao Zhang

**Affiliations:** 1 State Key Laboratory of Transducer Technology, Institute of Semiconductors, Chinese Academy of Sciences, Beijing 100083, China; tianmeng@semi.ac.cn (M.T.); mglou@semi.ac.cn (M.L.); hwzhu@semi.ac.cn (W.H.); 2 Center of Materials Science and Optoelectronic Engineering, University of Chinese Academy of Sciences, Beijing 100049, China; 3 Technical Institute of Physics and Chemistry, Chinese Academy of Sciences, Beijing 100190, China; zhangwei5260@outlook.com (W.Z.); yankaiqi@mail.ipc.ac.cn (K.Y.); binliao@mail.ipc.ac.cn (B.L.)

**Keywords:** strain and temperature, different temperature coefficient, femtosecond fiber Bragg grating

## Abstract

The temperature and strain fields monitoring during the preparation process of buoyancy materials, as well as the health status after molding, are important for mastering the mechanical properties of buoyancy materials and ensuring the safety of operators and equipment. This paper proposes a short and high-density femtosecond fiber Bragg grating (fs-FBG) array based on different temperature coefficients fibers. By optimizing the parameters of femtosecond laser point-by-point writing technology, high-performance fs-FBG arrays with millimeter level gating length and millimeter level spatial resolution were prepared on two types of fibers. These were successfully embedded in buoyancy materials to achieve in-situ online monitoring of the curing process and after molding. The experimental results show that the fs-FBG array sensor has good anti-chirp performance and achieves online monitoring of millimeter-level spatial resolution. Intelligent buoyancy materials can provide real-time feedback on the health status of equipment in harsh underwater environments. The system can achieve temperature monitoring with an accuracy of 0.56 °C and deformation monitoring with sub-millimeter accuracy; the error is in the order of micrometers, which is of great significance in the field of deep-sea exploration.

## 1. Introduction

Solid buoyancy material is a new type of composite material with high mechanical strength, low density, low water absorption, and good fatigue resistance, which is widely used in deep-sea exploration equipment. The performance of materials is related to the safety of equipment and staff. However, solid buoyancy materials require high temperature to promote curing, and the temperature distribution is uneven during the preparation process. The demolded specimens often have significant thermal residual strain, which can easily cause cracking and warping in high-pressure underwater environments, and affect the reliability of equipment [1]. Even the material is directly damaged during the preparation process, making it difficult to use in practical applications. Therefore, in order to prepare high-performance solid buoyancy materials and ensure the safety of equipment and workers, it is necessary to research an embedded array sensor that can monitor the temperature and strain distribution during the curing process of buoyancy materials in real time, as well as the structural state during use.

Fiber Bragg grating (FBG) sensors have been used in the curing process of many composite materials due to their small size, light weight, easy multiplexing, and good compatibility [2,3,4,5,6]. Although there have been some reports of FBGs used for monitoring the curing of buoyancy materials, the FBGs used are generally prepared using ultraviolet excimer laser phase mask technology, which requires removing the coating of fiber; the grating length is generally between 5 mm and 1 cm [7,8,9]. When subjected to non-uniform strain inside the material, there is no coating layer to buffer, and the reflection spectrum of FBGs with a longer grating length is prone to broadening and splitting [10]. Therefore, in order to achieve all-round and high-precision monitoring of buoyancy materials, it is urgent to develop a high-density, short-grating-length FBG array that does not require the coating to be removed. Femtosecond (fs) laser point-by-point (PbP) writing technology has prompted the rapid development of high-density FBG arrays due to its flexible and controllable center wavelength and grating length, as well as its ability to directly modulate the refractive index through the fiber coating. X. Z. Xu et al. [11] prepared an FBG array with a grating length of 4 mm and a physical spacing of 2 mm using fs-laser PbP writing technology. As the grating length of the FBG continues to decrease, the reflectivity also decreases while the 3 dB bandwidth increases, reaching several nm; this greatly increases the difficulty of demodulation and writing high-density FBG arrays. L. Q. Zhu et al. [12] used an 800 nm fs-laser PbP writing method to write an FBG array with a grating length of 3 mm and a spacing of 5 mm in a polarization-maintaining fiber, and two FBGs were multiplexed in a wavelength range of 40 nm. Y. P. Wang et al. [13] increased the refractive index modulation area by writing six FBGs with the same center wavelength at different radial positions on the same fiber segment, with a reflectivity of about 10% and a 3 dB bandwidth of about 9.4 nm. They used the same process to write FBGs with different center wavelengths. However, the 3 dB bandwidth was nearly 10 nm, and the reflectivity was also low, severely limiting the number of FBGs in dense wavelength division multiplexing (WDM) systems and making it difficult to achieve high-precision demodulation and high-density FBG arrays. It can be seen that FBGs with a long grating length can reduce the 3 dB bandwidth and improve reflectivity, but the physical length limits the size of the FBG array, which is not conducive to achieving high-resolution monitoring. FBGs with short grating length have a wide 3 dB bandwidth of and low reflectivity, which increases the difficulty of achieving dense wavelength division multiplexing. For buoyancy material specimens, it is required that the FBG does not exhibit chirp and spectrum broadening when subjected to non-uniform temperature and strain distribution inside the material, accurately monitoring the state changes at various positions of the buoyancy material. It is necessary to find a balance point between the length, reflectivity, and 3 dB bandwidth of the FBG to prepare high-density FBG arrays with a short grating length and narrow bandwidth.

Due to the simultaneous variation in temperature and strain fields during the curing of buoyancy materials, FBGs suffer from cross-sensitivity between temperature and strain. There are two methods to solve the problem: the temperature compensation method and the dual wavelength matrix method. The temperature compensation method introduces two identical FBGs and encapsulates one of the FBGs with a metal sleeve [14], a capillary glass tube [15], and a ceramic [16,17] to ensure that it is only affected by the temperature field. This solution is easy to operate and low-cost, but has poor compatibility. The intrusion of packaging tubes affects the mechanical properties of the material, introducing defects and damage [18]. The dual wavelength matrix method uses two FBGs with different temperatures or strain sensitivities. Both FBGs simultaneously sense the external temperature and strain, such as FBG and tilted fiber Bragg grating (TFBG) [19], FBG and long-period fiber grating (LPFG) [20], TFBG, and LPFG [21]. However, the length of the TFBG and LPFG is generally in the centimeter range, making it difficult to achieve a high-density array. It is also possible to construct a dual wavelength matrix using different types of fibers, such as a hollow-core fiber and a single-mode FBG fusion [22], a single-mode FBG and thin-core FBG fusion [23], or polarization maintaining a multi-mode fiber [24]. This method generally requires the fusion of fibers, which reduces the mechanical strength of the fibers. Therefore, it is of great significance to study a fiber Bragg grating array sensor that does not require packaging and can be directly embedded inside buoyancy materials to accurately measure the temperature and strain for buoyancy materials monitoring.

In this paper, to achieve high-density, short grating length, temperature and strain measurement of femtosecond fiber Bragg grating (fs-FBG) sensors, we propose a polyimide fiber Bragg grating and boron/germanium (B/Ge) co-doped fiber Bragg grating sensor array based on femtosecond laser point-by-point writing technology. By optimizing the fs-laser processing technology, FBG arrays with a grating length of 2 mm were successfully prepared on two types of fibers. Three FBGs were multiplexed at a wavelength interval of 12 nm, with a total array length of 22 mm, achieving a high-density and short-physical-length FBG array preparation. Without any packaging, they were directly embedded into buoyancy materials, successfully achieving temperature and strain monitoring at different positions during the curing process of buoyancy materials. The formed intelligent buoyancy material can sense the temperature and deformation.

## 2. Fabrication of Short Grating Length and High-Density fs-FBG Arrays Based on Different Temperature Coefficients

### 2.1. Fs-Laser Point-by-Point Writing Technology

**Figure 1:**
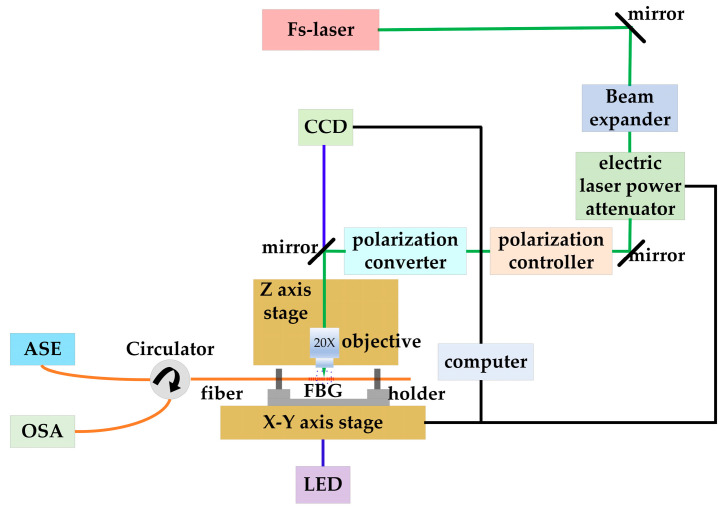
Schematic of the fs-laser PbP writing system.

The schematic of the fs-laser PbP writing system of the FBG arrays is shown in Figure 1. An fs laser system (light conversion, carbide) producing laser pulses with a duration of 220 fs at a central wavelength of 1030 nm is employed for inscribing. The laser maximum output power is 5 W and the repetition rate is adjustable within the range of 1 MHz. The fs laser is introduced into the Z axis stage after a beam shaping module, which is subsequently focused into the fiber core (YOFC) by a 20X objective (NA = 0.4). To eliminate the cylindrical astigmatism and positional distortion of the fiber during laser processing, the fiber is immersed in a refractive index-matching oil (Cargille, *n* = 1.4587). The average laser output power is adjusted by an electric laser power attenuator and quantitatively controlled in percentages through software. The movement of the three-dimensional (3D) translation stage is also controlled by the software of a computer, and the microstructure of FBG can be captured in real time through a CCD and an LED. It is monitored using an amplified spontaneous emission source (ASE) passing through a circulator and recording of either the reflection or transmission spectra with an optical spectrum analysis (OSA, APEX, AP2061A), and the resolution of the OSA is 1.12 pm.

The alignment between the laser spot and the fiber core is crucial for writing the FBG, which is carried out under the real-time observation of the CCD. Subsequently, we set the X axis stage to move along the fiber axis at a constant speed of *v* = 1.61 mm/s. Due to the pulse mode of the fs laser, the traces of the pulse at different positions in the fiber core can be obtained, and an FBG is fabricated. The central wavelength *λ_B_* of the FBG is [25]:

(1) $$m\lambda_{B}=2n_{eff}\Lambda =2n_{eff}v/f,$$

where *m* is the resonance order of the FBG; *n_eff_* is the effective refractive index of the fiber, Λ is the grating period, and *f* is the repetition rate of the laser. In the experiment, *m* = 3, *n_eff_* = 1.4475, *f* = 1 kHz, Λ is related to the central wavelength, and different wavelength FBGs can be fabricated by adjusting the processing speed, which reflects the flexibility of fs-laser PbP writing technology.

### 2.2. FBG Arrays Written by Fs-Laser in Different Fibers

**Figure 2:**
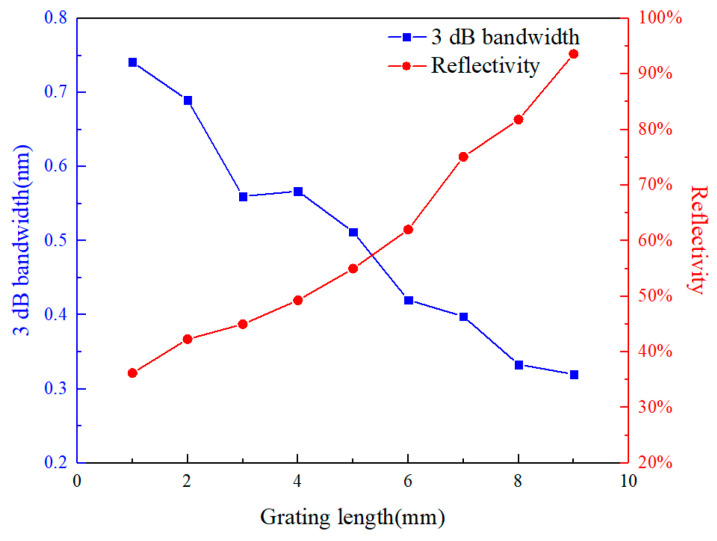
Variation in 3 dB bandwidth and reflectivity of FBG with grating length.

The key processing parameters of the fs-laser PbP writing technology were optimized to realize the short and high-density fs-FBG array. For FBGs, the reflectivity and 3 dB bandwidth are the main parameters that affect the sensing performance. The variation in FBG reflectivity and 3 dB bandwidth with a grating length of 1–9 mm was studied in the experiment. The single pulse energy was about 300 nJ, and there were five pulses, as shown in e Figure 2. It can be seen that the longer the fiber grating length is, the narrower the 3 dB bandwidth and the higher the reflectivity will be. However, an FBG with a long grating length is not conducive to achieving high-spatial-resolution monitoring. When the grating length was 1 mm, the reflectivity was less than 40%, which increased the difficulty of achieving high-precision demodulation. The reflectivity can be increased by increasing the depth of the refractive index modulation [26], but as the depth of the refractive index modulation increases, the 3 dB bandwidth also increases. Therefore, there is a contradictory relationship between the 3 dB bandwidth and reflectivity, and a balance point needs to be found.

Changing the single pulse energy and quantity of the fs-laser can achieve a change in the depth of the refractive index modulation, thereby creating fs-FBGs with different parameters [27].

**Figure 3:**
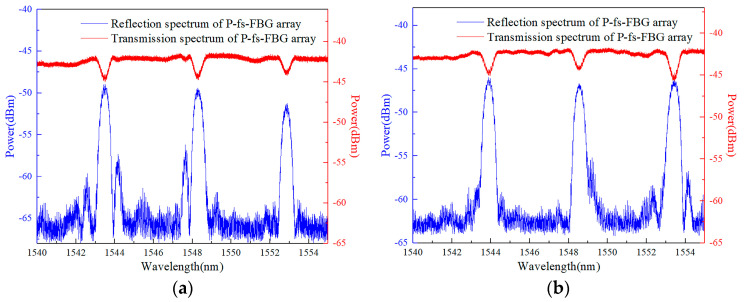
Spectra of the fs−FBG arrays: (**a**) polyimide fiber and (**b**) B/Ge co−doped fiber.

For the short fs-FBG required for buoyancy materials, in order to ensure a relatively high reflectivity, we controlled the grating length to 2 mm to ensure that there was no chirping due to an uneven strain field during the monitoring of the curing of buoyancy materials. The 3 dB bandwidth of the fs-FBG was also controlled within 1 nm, which was conducive to achieving a high-density fs-FBG array [28]. Through multiple process studies, it has been found that for polyimide fiber Bragg grating arrays, the energy of the fs laser single pulse was about 550 nJ, and there were 10 pulses per refractive index modulation point. For B/Ge co-doped fiber Bragg grating arrays, the energy of the fs laser single pulse was about 240 nJ, and there were 6 pulses per refractive index modulation point. The grating length of the fs-FBG was 2 mm, with an interval of 8 mm. Three FBGs are multiplexed in the wavelength range of 12 nm, with a total array length of approximately 22 mm. The reflection and transmission spectra of the fs-FBG array are shown in Figure 3, and it can be seen that the 3 dB bandwidth was about 0.5 nm, and the reflectivity *R* was about 50%. The reflectivity was calculated by the formula *R* = 1−10^−*T*/10^, where *T* was the transmission peak depth, which was about 3 dB [29]. The fs-FBG array with millimeter level grating length and millimeter level spatial resolution was fabricated, providing a core sensor component for intelligent buoyancy materials.

## 3. Temperature and Strain Monitoring of Buoyancy Materials Curing

### 3.1. Curing of Buoyancy Materials Based on fs-FBG Arrays

**Figure 4:**
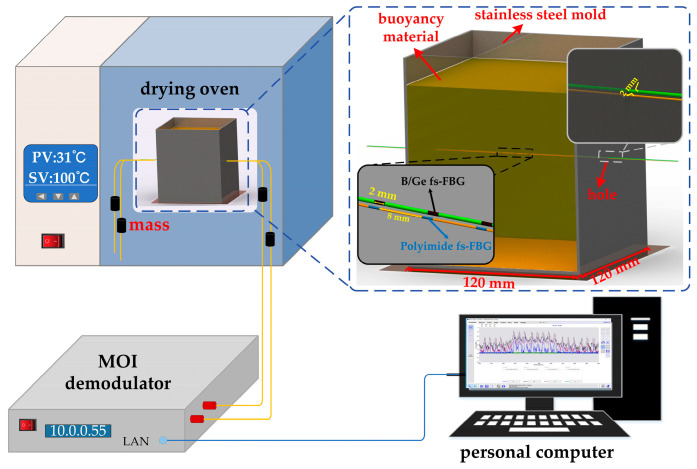
Experimental setup of buoyancy material curing monitoring based on fs-FBG arrays.

The experimental setup for monitoring the curing of buoyancy materials based on fs-FBG arrays is shown in Figure 4. The buoyancy materials were heated by an electric constant-temperature blast drying oven (DHG-9140AD) to promote curing. A set of fs-FBG arrays with different temperature coefficients were parallel embedded inside the buoyancy materials, and the center wavelength of the fs-FBG array was recorded in real time by an MOI demodulator (LUNA, si155). The center wavelength change Δ*λ_B_* was related to the temperature and strain [30]:

(2) $$\frac{\Delta \lambda_{B}}{\lambda_{B}}=K_{T}\Delta T+K_{\varepsilon }\Delta \varepsilon ,$$

where *λ_B_* is the center wavelength, *K_T_* is the temperature sensitivity, Δ*T* is the temperature change, *K_ε_* is the strain sensitivity, and Δ*ε* is the strain change. The response of two fs-FBG arrays could be expressed:

(3) $$\left(\begin{array}{c} {\Delta \lambda }_{B1}\\ {\Delta \lambda }_{B2} \end{array}\right)=\left(\begin{array}{cc} K_{T1} & K_{\varepsilon 1}\\ K_{T2} & K_{\varepsilon 2} \end{array}\right)\left(\begin{array}{c} \Delta T\\ \Delta \varepsilon \end{array}\right),$$

where Δ*λ_B_*_1_ is the center wavelength change of polyimide fs-FBG, *K_T_*_1_ and *K_ε_*_1_ are the temperature and strain sensitivities, respectively; Δ*λ_B_*_2_ is the central wavelength change of B/Ge co-doped fs-FBG, *K_T_*_2_ and *K_ε_*_2_ are the temperature and strain sensitivities, respectively. Thus, by solving the inverse matrix of Equation (2), both values could be obtained from the central wavelengths [31,32]:

(4) $$\left(\begin{array}{c} \Delta T\\ \Delta \varepsilon \end{array}\right)=\frac{1}{M}\left(\begin{array}{cc} K_{\varepsilon 2} & {-K}_{\varepsilon 1}\\ {-K}_{T2} & K_{T1} \end{array}\right)\left(\begin{array}{c} {\Delta \lambda }_{B1}\\ {\Delta \lambda }_{B2} \end{array}\right)$$

where *M* = *K_T_*_1_*K_ε_*_2_ − *K_T_*_2_*K_ε_*_1_ is the matrix determinant.

**Figure 5:**
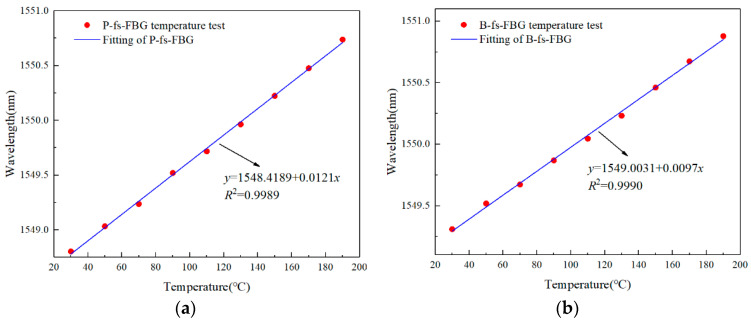

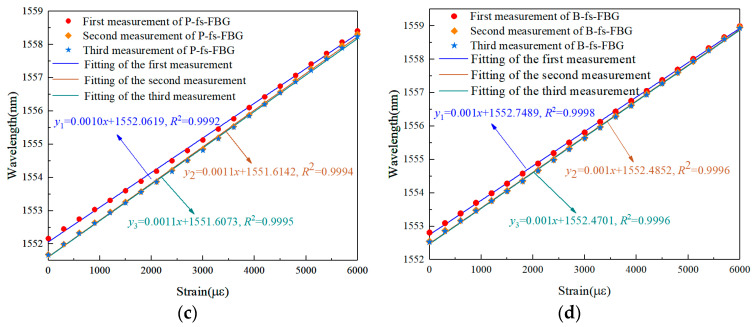
Temperature sensitivity calibration: (**a**) polyimide fs-FBG and (**b**) B/Ge co-doped fs-FBG; Strain sensitivity calibration: (**c**) polyimide fs-FBG and (**d**) B/Ge co-doped fs-FBG.

Through temperature and strain sensitivity calibration, the temperature sensitivity *K_T_*_1_ and *K_T_*_1_ of polyimide fs-FBG (P-fs-FBG) and B/Ge co-doped fs-FBG (B-fs-FBG) in the range of 30–170 °C were approximately 12.1 pm/°C and 9.7 pm/°C, respectively; and both types of fs-FBGs had a strain sensitivity (*K_ε_*_1_ and *K_ε_*_2_) of 1.0 pm/με, as shown in Figure 5. Three experiments were conducted to calibrate the strain sensitivity to better ensure the accuracy of the measurement results and reduce errors. Therefore, Equation (4) can be expressed as:

(5) $$\left(\begin{array}{c} \Delta T\\ \Delta \varepsilon \end{array}\right)=\frac{1}{2.4}\left(\begin{array}{cc} 1 & -1\\ -9.7 & 12.1 \end{array}\right)\left(\begin{array}{c} {\Delta \lambda }_{B1}\\ {\Delta \lambda }_{B2} \end{array}\right)$$

The measurement results of polyimide fs-FBG and B/Ge co-doped fs-FBG indicated that they overcame the problem of strain and temperature cross-sensitivity in traditional FBGs, and could achieve measurements of temperature and strain in principle by utilizing different temperature sensitive properties. The temperature monitoring resolution of the fs-FBG can be expressed by the formula [18]:

(6) $$∆T=\frac{∆\lambda }{\left|K_{T1}-K_{T2}\right|}$$

where, Δ*T* is the temperature resolution and Δ*λ* is the wavelength resolution of the fiber Bragg grating demodulator. By using fiber sensors with significantly different sensitive characteristics, a higher temperature resolution can be achieved. In the experiment, the wavelength resolution of the MOI demodulator used is 1 pm, so the temperature monitoring resolution is about 0.42 °C.

When fs-FBG arrays were used for monitoring the curing of buoyancy materials, to ensure that the temperature and strain fields perceived by the two fs-FBG arrays were the same, polyimide fs-FBG array and B/Ge fs-FBG array were placed side by side in the center of the mold with a spacing of 2 mm, to ensure that the perceived external environment was essentially the same. Two fs-FBG arrays were directly embedded in the buoyancy material, and a prestrain of 642 με was applied at both ends to make sure the sensor was in a working condition.

**Figure 6:**
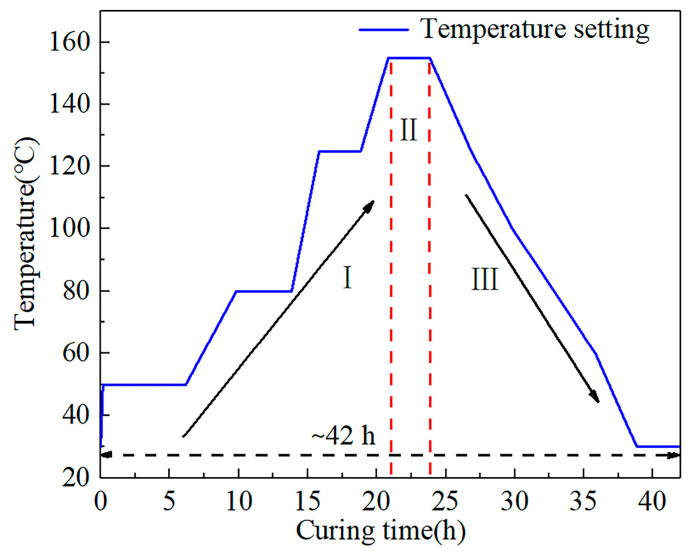
Curing process temperature setting.

The buoyancy materials were provided by the Technical Institute of Physics and Chemistry, Chinese Academy of Sciences, and consisted of two components, hollow glass beads and epoxy resin. The average particle size of the hollow glass beads was about 40 μm. The curing process, which included three steps, is shown in Figure 6: heating, insulation, and cooling. The initial temperature was 30 °C, and after four rounds of heating to 155 °C to ensure that the buoyancy material was fully cured, it was subsequently slowly cooled down to 30 °C. The curing process lasted approximately 42 h.

### 3.2. Fs-FBG Array in Buoyancy Materials after Curing

**Figure 7:**
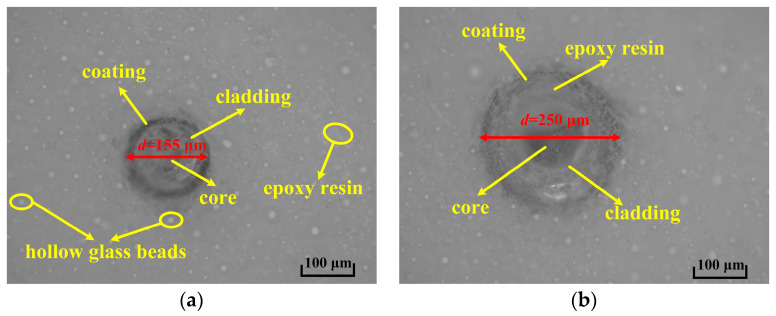
Cross-sectional optical microscopy: (**a**) polyimide fs-FBG; (**b**) B/Ge co-doped fs-FBG.

After 42 h of the curing process, the buoyancy material was formed. The sample areas with a high strain measured by the different fs-FBGs were observed using an optical microscope, and the results are shown in Figure 7. After the buoyancy material was formed, both types of fs-FBGs were well coupled with the buoyancy material, and no abnormal situations were observed in the coating, cladding, and fiber core. In an environment of thousands of με, the fs-FBGs survived perfectly. From Figure 7, it can also be seen that fs-FBG sensors can be well combined with the substrate material, maintaining good consistency and fully demonstrating the advantage of the fiber optic sensor scheme.

**Figure 8:**
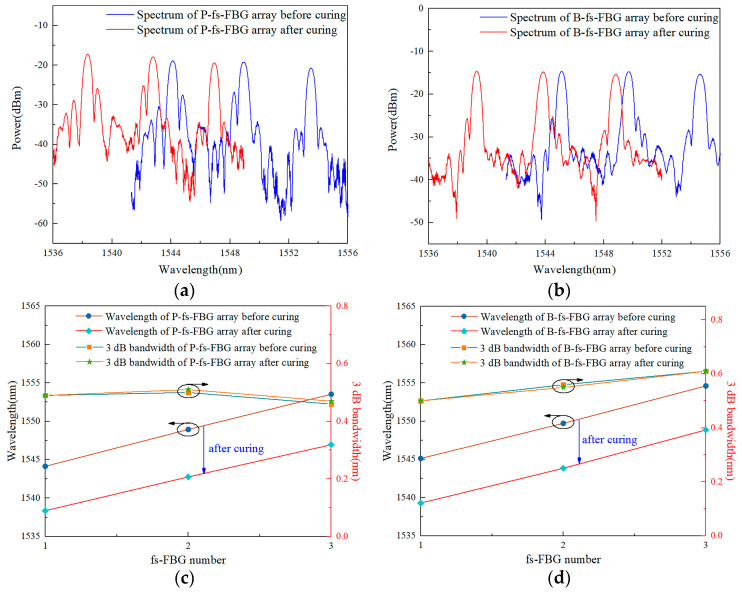
Fs−FBG arrays reflection spectra: (**a**) polyimide fiber and (**b**) B/Ge co−doped fiber; wavelength and 3 dB bandwidth changes: (**c**) polyimide fiber and (**d**) B/Ge co−doped fiber.

The reflection spectra of embedded fs-FBG arrays before and after the curing process are presented in Figure 8. The center wavelengths of the fs-FBG sensors moved toward the shortwave direction after curing, and the fs-FBG arrays had good waveforms and symmetry at the end of the curing process. Figure 8c,d shows the changes in the center wavelength and 3 dB bandwidth of the fs-FBG arrays before and after curing. Due to the different temperature sensitivity of polyimide and B/Ge co-doped fs-FBGs, the wavelength shift of the two fs-FBG arrays was not the same. Both sensors did not exhibit spectral degradation phenomena such as chirp distortion and spectral broadening.

### 3.3. Temperature and Strain Curing Curve Analysis of Buoyancy Material

**Figure 9:**
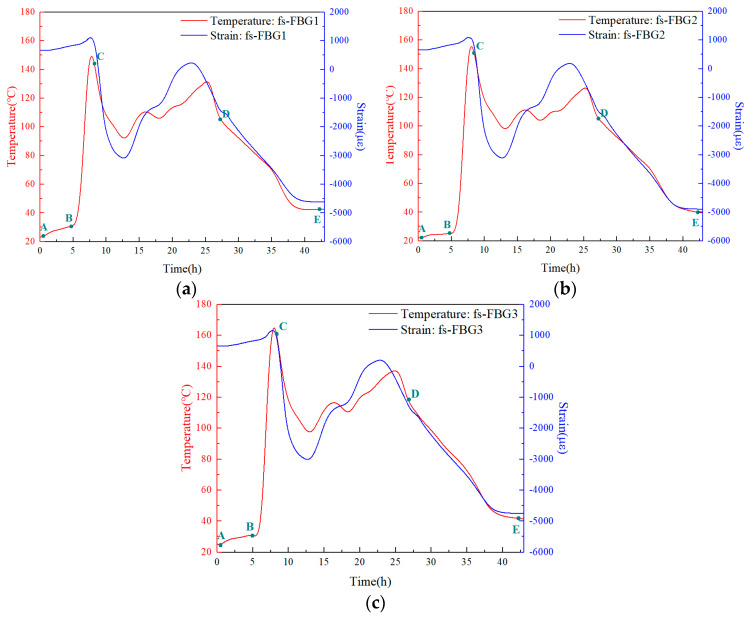
Temperature and strain curing curve analysis of buoyancy material: (**a**) fs−FBG1; (**b**) fs−FBG2; (**c**) fs−FBG3.

The buoyancy material was placed in an electric constant-temperature blast drying oven and cured according to the process shown in Figure 6. According to Equation (5), the temperature and strain distribution during the curing process of the buoyancy material monitored by two types of fs-FBG arrays are shown in Figure 9. This was similar to the monitoring results of the fiber Bragg grating fabricated by our previous ultraviolet excimer laser phase-mask technology [7], but compared to before the research, the curing process has been changed, so the temperature and strain monitoring curves were different. Moreover, in this study, the removal of capillary glass tubes ensured a high consistency of the solid buoyancy material, increased the compressive strength of the buoyancy material, and avoided the rupture of capillary glass tubes under high pressure, which would affect the reliability of the material. We conducted a detailed analysis of the curing process of the buoyancy material in several stages. In the early stage of the curing process (A-B period), the resin crosslinking reaction inside the buoyancy material had not yet begun and was still in a viscous state. Therefore, the temperature perceived by the fs-FBG array was the result of external heat transfer. Due to the poor thermal conductivity of the buoyancy material, the temperature was approximately linearly increased, and the strain magnitude remained at pre-strain.

In the subsequent B-C stage, as the temperature increased the buoyancy material continuously absorbed heat and the intramolecular energy increased. The input of external energy intensified the thermal motion and intermolecular collisions of molecules, and the resin began complex physical and chemical reactions. At this stage, the resin released a large amount of heat, which was perceived by the fs-FBG array sensor. The highest peak heat release measured by fs-FBG3 was 165 °C, while the temperature measured by fs-FBG1 was 149 °C. This indicated that for every 16 mm position change, the temperature changes by about 16 °C, and the internal temperature distribution of the material is uneven. The viscosity of the buoyancy material increased with the increase in the curing degree, gradually transforming from a fluid to a viscoelastic solid with a certain hardness. Due to the uneven curing degree at various positions, internal stress and curing deformation were generated.

In the C-D stage, also known as the gel stage [33], after prolonged heating, the buoyancy material underwent sufficient curing reactions. The non-uniform temperature and strain fields were measured by fs-FBG array, and the central wavelength of the fs-FBG array moved to the short wavelength direction [34]. In the final D-E stage, the high viscosity of the material increased the internal friction and hindered the progress of chemical reactions, which were mainly the physical crosslinking processes [35]. At this point, the shrinkage strain reached its maximum, approximately −4891 με, marking the end of curing. Combined with Figure 8, it can be seen that both fs-FBG array spectra remained integral in thousands of με environments. The experimental results indicated the advanced nature of the sensor. Two kinds of fs-FBG arrays with different temperature coefficients could separate the temperature and thermal residual strain information during the curing process of buoyancy materials, realize the measurement of the temperature and strain, and play an auxiliary role in mastering the mechanical properties of the solid buoyancy materials.

### 3.4. Intelligent Buoyancy Material Temperature and Strain Sensing

**Figure 10:**
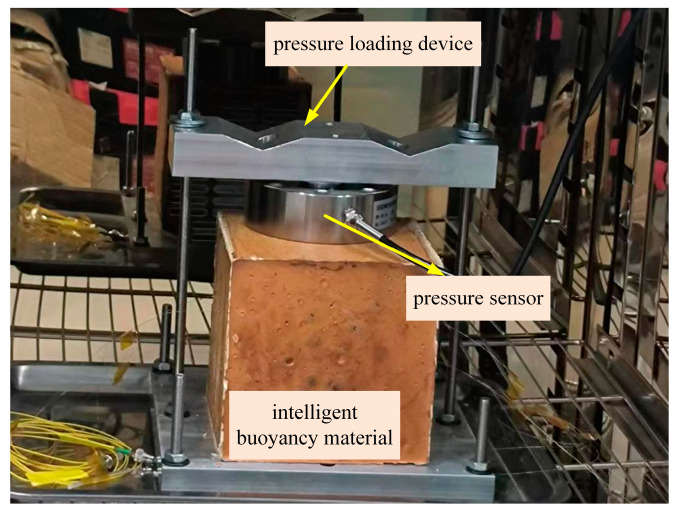
Schematic diagram of pressure loading and monitoring.

After 42 h of a curing reaction, the femtosecond fiber Bragg grating has been integrated with the buoyancy material, becoming an intelligent buoyancy material that can achieve self-perception and self-adjustment. Temperature and strain are two of the key factors affecting the reliability of intelligent buoyancy materials. Real-time monitoring of the perceived temperature and strain of buoyancy materials provides a reference for adjusting engineering operations and ensures the performance of buoyancy materials under various environmental conditions. Buoyancy materials were applied at different temperatures and strains to verify their self-sensing temperature and strain capacity. In the experiment, the intelligent the strain of the buoyancy material was applied through a pressure-loading device. Because the intelligent buoyancy material was an isotropic medium, the applied pressure was directly proportional to the deformation and strain. The applied pressure measured by the pressure sensor was converted to the deformation and strain of the intelligent buoyancy material. The Poisson’s ratio of the buoyancy material was 0.3 and the elastic modulus was 4 GPa. To verify the accuracy and reliability of the data measured by the intelligent buoyancy material, a pressure sensor was also fixed on the pressure application device to provide a pressure reference value, as shown in Figure 10.

Due to the fact that fs-FBGs were integrated with solid buoyancy materials, both the temperature and strain sensitivity were affected by the buoyancy materials. When the temperature changed, the fs-FBG directly contacted the buoyancy material, which was not only affected by the temperature, but also by the thermal expansion effect of the solid buoyancy material. When the temperature changed Δ*T*, the strain generated on fs-FBG could be expressed as [36,37]:

(7) $$\varepsilon_{T}=\left(\alpha_{sub}-\alpha \right)\Delta T,$$

where *α_sub_* was the thermal expansion coefficient of the buoyancy materials; in the experiment about 4 × 10^−5^/°C, α was the thermal expansion coefficient of fibers, usually 5.5 × 10^−7^/°C [38,39], where *α_sub_* was much larger than α. Therefore, the impact of temperature changes on the center wavelength of fs-FBG was:

(8) $$\Delta \lambda_{B}=\left[\xi +\left(1-P_{e}\right)\alpha_{sub}\right]\Delta T\cdot \lambda_{B}$$

where *ξ* was the thermal optical coefficient of fs-FBG, generally 8.3 × 10^−6^/K, *P_e_* was the effective elastic coefficient, generally taken as 0.22 [40]. Therefore, the temperature sensitivity coefficient of fs-FBG could be expressed as *K_T_*’ = [*ξ* + (1 − *P_e_*)*α_sub_*] ≈ 3.95 × 10^−5^/°C.

To more accurately obtain the sensing ability of the two types of fs-FBGs embedded in buoyancy materials, we calibrated the temperature and strain of the intelligent buoyancy material. The temperature sensitivity of polyimide fs-FBG and B/Ge co-doped fs-FBG was 45.1 pm/°C and 42.9 pm/°C, respectively. The difference of 0.2 pm/°C was mainly due to the normalization effect after coupling the fs-FBG with the buoyancy material. The strain sensitivity was 0.504 pm/με and 0.463 pm/με, respectively. It can be seen that the strain sensitivity decreased compared to the bare fs-FBGs. This was because the stiffness of buoyancy materials was relatively higher than the fiber.

**Figure 11:**
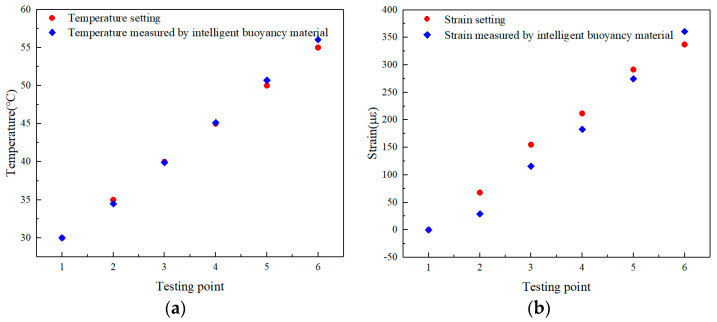
(**a**) Temperature and (**b**) strain measured by intelligent buoyancy material.

The temperature test range was 30~55 °C, and the strain test range was 0~340 με. Considering the poor thermal conductivity of buoyancy materials, each temperature point was stable for 4 h to ensure temperature transfer to the interior of the buoyancy materials. The temperature and strain experimental results are shown in Figure 11. It can be seen that when the temperature and strain changed, the intelligent buoyancy material could separate two types of signals, with temperature and strain monitoring accuracies of 0.56 °C and 28 με, respectively. The monitoring accuracy had decreased due to the fact that the temperature and strain perceived by the fs−FBG embedded in the buoyancy material were transmitted by the buoyancy material. The experimental results indicate that the intelligent buoyancy material has the ability to perceive the external temperature and strain, which provides a new monitoring method for material condition and equipment safety evaluation.

### 3.5. Intelligent Buoyancy Material Pressure Sensing

**Figure 12:**
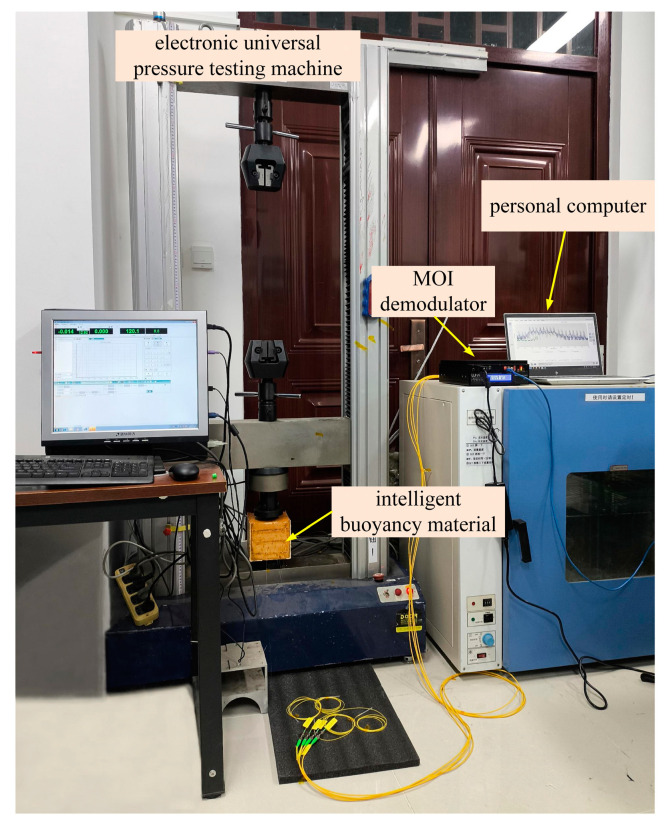
Compression experimental of intelligent buoyancy material.

Solid buoyancy materials are mainly used in high-pressure underwater environments. Therefore, pressure is another major factor affecting the performance of buoyancy materials. In this study, the working environment of intelligent buoyancy materials was simulated by applying pressure to evaluate their performance under different pressures. The intelligent buoyancy material was fixed on an electronic universal pressure testing machine (Jinan Dongce Testing Machine Technology Co., Ltd., Jinan, China) and the repeatability and reliability of fs-FBG monitoring were verified through two tests, as shown in Figure 12. The compression degree of intelligent buoyancy materials was 1%. Due to the size of the buoyancy material being 120 mm × 120 mm × 128 mm, the compression height was set to 1.28 mm. The electronic universal pressure testing machine was set up by the computer to slowly apply pressure at a speed of 0.256 mm/min, maintain 300 s at the position of compression to 1.28 mm, and then slowly moved upward at a speed of 0.256 mm/min until the intelligent buoyancy material was in a free state. The entire test lasted for about 16 min, and the external ambient temperature was maintained at 22 °C.

**Figure 13:**
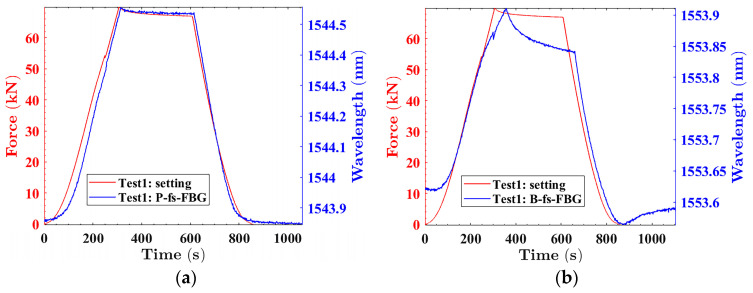

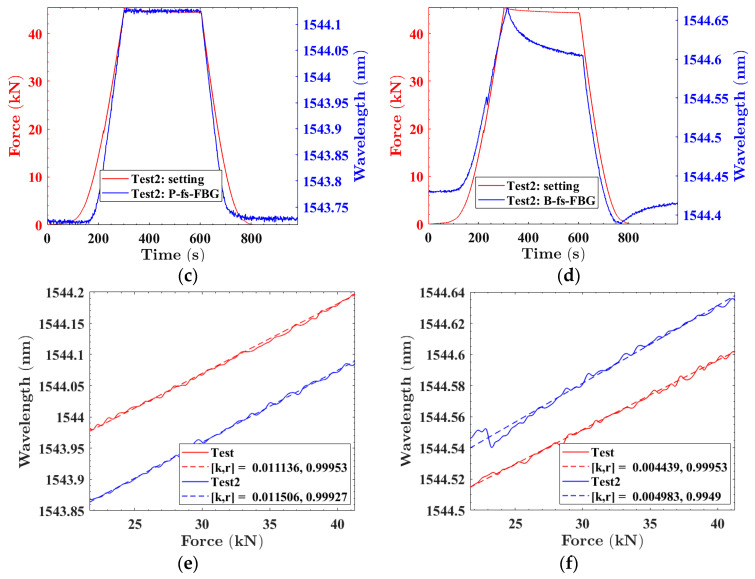
Pressure test of solid buoyancy material: (**a**,**b**) were the results of the first test of polyimide fs-FBG and B/Ge fs-FBG; (**c**,**d**) were the second test; (**e**,**f**) were the relationship between polyimide fs-FBG and B/Ge fs-FBG with applied pressure, respectively.

The second fs-FBG of two types of fs-FBG arrays was selected for analysis, as shown in Figure 13a–d. The center wavelength of the femtosecond fiber Bragg grating changes in real time with the applied pressure. From the red curves in Figure 13a,c, it can be seen that although the compression deformation applied twice was 1%, the maximum pressure provided by the electronic universal pressure-testing machine was different, which might be caused by the equipment as well as by human error. In the experiment, we determined the position where the electronic universal pressure-testing machine just came into contact with the buoyancy material as the initial point, which was compressed downwards by 1.2 mm. Due to the difference in the position of the initial point, the deformation was different, resulting in different pressures. Figure 13a,c shows the results obtained from polyimide fs-FBG measurements, while Figure 13b,d shows the results obtained from B/Ge co-doped fs-FBG. It can be seen that polyimide fs-FBG had a higher response accuracy. However, there were differences between the pressure changes measured by B/Ge co-doped fs-FBG and the settings. This was mainly due to the different coating types of the two types of fs-FBGs. The polyimide coating has strong adhesion and a better strain transfer performance, which can accurately reflect external pressure changes, while the coating layer of B/Ge co-doped fiber is acrylic resin, which is not tightly adhered to the fiber-core-like polyimide. When subjected to pressure, its mechanical properties are not as good as polyimide polymer materials. Therefore, the response to pressure was not sensitive enough, and there was a certain hysteresis effect. The experimental results showed that the polyimide fs-FBG had a higher response accuracy. Due to the constant temperature, choosing a polyimide fs-FBG for pressure testing can meet the requirements. In the future, the doping concentration of B/Ge co-doped fibers can be increased to improve the accuracy of temperature and strain monitoring. At the same time, the coating layer of B/Ge co-doped fibers is also changed to polyimide to increase the adhesion between the coating layer and the fiber core, ensuring that the coating layer can tightly adhere to the fiber core during the buoyancy material curing process and after curing, reducing hysteresis effects and improving monitoring accuracy.

The relationship between the sensor and the pressure is shown in Figure 13e,f. The sensitivity of the two measurements of the polyimide fs-FBG and the B/Ge fs-FBG was similar, with a difference of 0.37 pm/kN and 0.544 pm/kN, respectively, and the linearity was good. This indicated that the intelligent buoyancy material embedded in the fs-FBG array could provide real-time feedback on changes in external pressure, and the stability of fs-FBGs was good, with sensitivity remaining relatively stable under different pressures.

**Figure 14:**
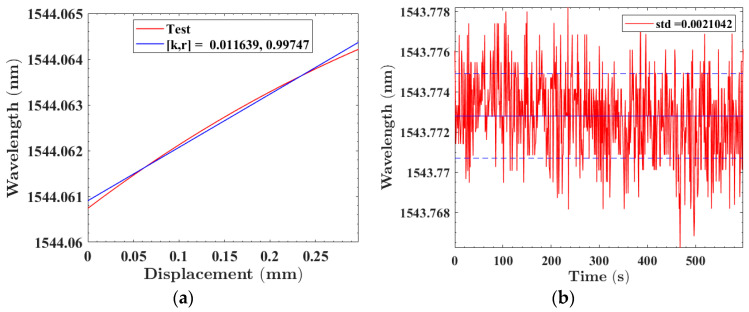
(**a**) Calibration of displacement sensitivity of intelligent buoyancy materials based on fs-FBG; (**b**) fs-FBG wavelength standard deviation within 10 min.

**Figure 15:**
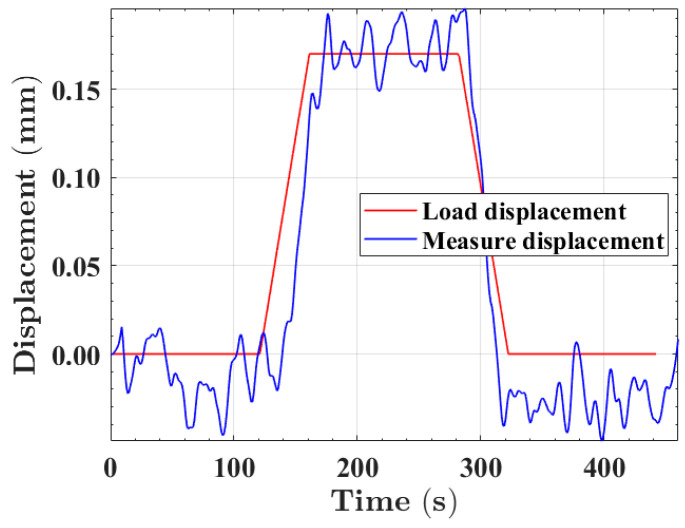
Intelligent buoyancy material displacement measurement.

By applying different pressures, the deformation of intelligent buoyancy materials could be obtained. Polyimide fs-FBG2 was selected to analyze the deformation monitoring capability of the intelligent buoyancy material system. The identifiable minimum displacement of the system can be represented as ΔX = *λ_std_*/*K*, where *λ_std_* is the standard deviation of the fs-FBG center wavelength, and *K* is the displacement sensitivity of the fs-FBG. By calibrating the displacement of intelligent buoyancy materials, the sensitivity could be determined to be 12 pm/mm, as shown in Figure 14a. Figure 14b shows the wavelength standard deviation of the fs-FBG within 10 min, which was approximately 2 pm. Therefore, the minimum displacement that the fs-FBG could recognize was about 0.17 mm. By controlling the universal pressure testing machine to apply a displacement of 0.17 mm to the intelligent buoyancy material at a speed of 0.256 mm/min, and maintaining it for 120 s, then returning to the original position at a speed of 0.256 mm/min. As can be clearly seen from Figure 15, the system could accurately identify a deformation of 0.17 mm. However, due to the fluctuation in the fs-FBG center wavelength detected by the MOI demodulator, the average measurement data within 120 s were taken to be 0.164 mm, with an error of 0.006 mm. By conducting compression tests on intelligent buoyancy materials, it can be found that fs-FBGs can sense external pressure in real time. When intelligent buoyancy materials are subjected to significant external impacts, resulting in material deformation and splitting, fs-FBG array sensors can accurately monitor them and ensure the reliability of the equipment.

## 4. Conclusions

In this paper, we proposed a short fs-FBG array based on different temperature coefficients fiber for monitoring the temperature and strain of intelligent buoyancy materials. By optimizing the processing parameters of fs laser, short and high-density fs-FBG arrays with a millimeter-level grating length and a millimeter-level spatial resolution were successfully prepared. Directly embedded inside the buoyancy material, the curing process of the intelligent buoyancy material and in-situ online monitoring after molding were successfully achieved. The fs-FBG array sensor maintained good spectral performance during the monitoring process without a significant chirp and broadening phenomenon, and the data reliability and accuracy were high. The fs-FBG array sensor is of great significance for achieving in situ online monitoring of intelligent buoyancy materials in harsh underwater environments.

## Data Availability

The data are not publicly available due to the Confidentiality and Non-disclosure Agreement with the funders.
